# Tooth loss and mortality risk: the mediating role of hs-CRP in a Chinese cohort

**DOI:** 10.3389/froh.2025.1542147

**Published:** 2025-04-24

**Authors:** Donglei Wu, Mingxin Mao, Wei Wang, Henan Zheng, Hongxia You, Weixuan Chen, Ziyang Xu, Yuyan Zheng, Li Yuan

**Affiliations:** Department of Stomatology, Shenzhen People’s Hospital, Shenzhen, China

**Keywords:** tooth loss, mortality, C-reactive protein, mediation analysis, retrospective cohort study

## Abstract

**Objectives:**

This study aimed to evaluate the mediating effect of systemic condition on the relationship between tooth loss and mortality risk.

**Materials and methods:**

A 9-y follow-up prospective longitudinal study was conducted based on China Health and Retirement Longitudinal Study (CHARLS). The participants aged >45 y at baseline and were followed up from 2011 to 2020. Cox proportional hazards models were utilized to assess the relationship between tooth loss and both all-cause mortality with hazard ratios (HRs) and 95% confidence intervals (CIs) reported with adjusted possible confounders. Systemic inflammation markers, including high-sensitivity C-reactive protein (hs-CRP) and white blood cell count (WBCs), were collected from CHARLS blood sample data. A mediation analysis was conducted to determine the role of hs-CRP and WBCs in the relationship between tooth loss and mortality.

**Results:**

A total of 13,201 participants met the inclusion criteria, of which 964 had tooth loss and 12,237 did not. During a median follow-up of 8.7 years, The multivariable-adjusted Cox regression models. The subgroup analysis indicated that the association was found to be stronger among older adults (≥80 years) (HR: 1.62, 95% CI: 1.09–2.41) and males (HR: 1.80, 95% CI: 1.34–2.40). Additionally, the mediation analysis result has showed that serum hs-CRP level rather WBC count mediated 3% of this effect.

**Conclusions:**

Complete tooth loss is associated with higher mortality in the Chinese population, with systemic inflammation (hs-CRP) as a mediator.

## 1 Introduction

Tooth loss is a prevalent and multifactorial condition in both developed and developing countries, with significant implications for individual health and quality of life. The Global Burden of Disease (GBD) study reported 267 million cases of tooth loss in 2017, with an age-standardized prevalence of 3.3% (1). The primary etiologies of tooth loss include dental caries and periodontal diseases, dental caries, and traumatic injuries, with the contribution of these factors varying by age group and population (2, 3).The implications of tooth loss are profound, affecting both oral function and systemic health (4).

The loss of teeth leads to diminished masticatory efficiency, which can compromise nutrition due to difficulty in consuming a balanced diet (5). Moreover, tooth loss is associated with bone resorption in the jaws, leading to altered facial aesthetics and structural integrity. Beyond local effects, tooth loss has been correlated with systemic conditions, including cardiovascular diseases, through pathways involving chronic inflammation and infection. The link between tooth loss and systemic conditions has been well established. Older individuals and males were significantly more likely to belong to the periodontitis group and exhibited a higher number of missing teeth (6, 7). Previous studies have demonstrated that participants with fewer teeth had experienced higher rates of all-cause and disease-specific mortality, including mortality from heart disease and diabetes mellitus (DM) (8).

However, the specific mechanism of the association between tooth loss and all-cause mortality was limited. Recent research has found that frailty status mediated the association the association between tooth loss and mortality (9). Since systemic inflammatory biomarkers such as C-reactive protein (CRP) and white blood cell (WBC) counts are associated with both tooth loss (10, 11) and all-cause mortality (12, 13), we hypothesized that systemic inflammation mediates the relationship between tooth loss and mortality. Therefore, Hence, the primary aim of this study was to investigate the association between tooth loss and all-cause mortality in a retrospective cohort of the Chinese population. A secondary aim was to determine the role of systemic inflammation marker as a mediator of this association.

## 2 Material and methods

### 2.1 Data source

Data for this study was obtained from the China Health and Retirement Longitudinal Study (CHARLS) database. CHARLS is a nationally representative longitudinal survey targeting Chinese residents aged 45 years and older. The survey is structured to collect comprehensive data on various aspects of the aging population in China, including demographics, family structure, health status, physical functioning, lifestyle behaviors, economic conditions, access to healthcare, and psychological well-being. The CHARLS dataset is publicly available to researchers worldwide. Ethical approval for the survey was granted by the Biomedical Ethics Committee of Peking University (IRB00001052-11015), and informed consent was obtained from all participants through signed consent forms.

### 2.2 Study design

This study utilized data from the 2011 baseline and 2020 follow-up surveys of the CHARLS, which included participants aged over 45 years from 450 villages, 150 counties, and 28 provinces. The exclusion criteria were as follows: (1) age under 45 years, and (2) missing information on tooth loss or all-cause mortality.

### 2.3 Exposure assessment

Tooth loss exposure was defined as self-reported complete edentulism, determined through the 2011 baseline community health survey under the general health status and disease history section. Using a structured questionnaire administered at enrollment, participants were asked to respond to the validated screening item: “Have you experienced complete loss of all natural teeth?” Responses were recorded in binary format (yes/no).

### 2.4 Outcome ascertainment

The primary endpoint for this study was all-cause mortality, verified through standardized verbal autopsy protocols between baseline assessment (2011) and study termination (2020). Mortality statue was documented via verbal autopsy and coded as a binary variable representing death or alive.

### 2.5 Potential confounders

Based on prior knowledge, several potential confounders were incorporated into the model adjustments, including demographic characteristics, lifestyle factors, and health status. Demographic variables included age, sex (male or female), marital status (married or other), place of residence (rural or urban), and body mass index (BMI). Lifestyle factors comprised smoking status (yes or no) and alcohol consumption (yes or no). Health status was evaluated through the self-reported history of systemic diseases, including hypertension, dyslipidemia, diabetes mellitus (DM), and cardiovascular disease (CVD).

### 2.6 Laboratory analysis

Systemic inflammation markers, including high-sensitivity C-reactive protein (hs-CRP, mg/dl) and white blood cell count (WBCs, thousand/μl), were obtained from CHARLS 2011 blood sample data. Venous blood samples were collected from participants and processed via centrifugation to separate plasma and buffy coat. The hs-CRP levels were measured using an immunoturbidimetric assay at the Youanmen Center for Clinical Laboratory of Capital Medical University. Complete blood count (CBC) analysis, which includes white blood cell count measurements, was performed at local Centers for Disease Control (CDC) laboratories.

### 2.7 Statistical analysis

Age and BMI were summarized as median values with interquartile range (IQR) for continuous variables. Categorical variables, including sex, marital status, residence, drinking and smoking status, and medical history (hypertension, DM, CVD, and dyslipidemia) were presented as counts and percentages. Comparisons of continuous variables with non-normal distributions between the tooth loss and non-tooth loss groups were performed using the Mann–Whitney *U* test, while categorical variables were compared using the Chi-squared test. Missing data for covariates were addressed through multiple imputation using the “mice” package in R software.

Cox proportional hazards models were employed to assess the relationship between tooth loss and all-cause mortality. Results were reported as with hazard ratios (HRs) and 95% confidence intervals (CIs). Three models were established: Model 1 adjusted for sex, age, marital status and residence, Model 2 further adjusted for BMI, alcohol consumption and smoking status in addition to the variables in Model 1. Model 3 included all variables from Model 2 and additionally adjusted for hypertension, DM, CVD, and dyslipidemia.

A mediation analysis was also conducted to explore whether systemic inflammation markers mediate the relationship between tooth loss and mortality. This analysis utilized the “mediation” package in R software (version 4.1), with the Bootstrap method applied to estimate the standard error of the mediation effect, thereby enhancing the robustness of the results. Two main effects were analyzed: average causal mediation effect (ACME) and the average direct effect (ADE). The ACME represents the indirect effect of tooth loss on mortality through systemic inflammation markers, whereas the ADE reflects the direct effect of tooth loss on mortality, independent of systemic inflammation markers. Statistical significance was determined with a *p*-value threshold of less than 0.05.

## 3 Results

### 3.1 Baseline characteristics of participants based on tooth loss

A total of 13,201 participants met the inclusion criteria, of whom 964 had tooth loss and 12,237 did not. Among the participants, 21% had missing covariate data. Specifically, missing values were observed for 20.3% of BMI, 0.06% of sex, 0.13% of hypertension, 0.24% of DM, 0.63% of dyslipidemia, 0.21% of CVD, 0.05% of alcohol consumption, and 0.02% of smoking status. All the missing data was handled using multiple imputation.

Table 1 summarizes the baseline characteristics of participants based on tooth loss. This cohort comprised 6,252 males (47.36%) and 6,949 females (52.64%) with a mean age of 58 years. Participants with tooth loss were generally older, more likely to be female, had lower body weight, lived in rural areas, and had a higher mortality and higher prevalence of hypertension and CVD (*p* < 0.05).

**Table 1:** Baseline characteristics of participants based on tooth loss.

Variables	Total (*n* = 13,201)	Without tooth loss (*n* = 12,237)	Tooth loss (*n* = 964)	*p* value
BMI	23.49 ± 3.94	23.60 ± 3.94	22.21 ± 3.74	<0.001
Age	58.19 ± 9.06	57.44 ± 8.60	67.73 ± 9.34	<0.001
Death age	73.23 ± 10.67	71.25 ± 10.53	79.55 ± 8.43	<0.001
Follow-up years	8.74 ± 1.37	8.78 ± 1.24	8.17 ± 2.38	<0.001
Death				<0.001
No	12,722 (96.37)	11,872 (97.02)	850 (88.17)	
Yes	479 (3.63)	365 (2.98)	114 (11.83)	
Sex				<0.01
Female	6,949 (52.64)	6,393 (52.24)	556 (57.68)	
Male	6,252 (47.36)	5,844 (47.76)	408 (42.32)	
Marital status				0.48
Other	267 (2.02)	244 (1.99)	23 (2.39)	
Married	12,934 (97.98)	11,993 (98.01)	941 (97.61)	
Residence place				<0.001
Rural	8,412 (63.72)	7,711 (63.01)	701 (72.72)	
Urban	4,789 (36.28)	4,526 (36.99)	263 (27.28)	
Hypertension				<0.001
No	8,469 (64.15)	7,916 (64.69)	553 (57.37)	
Yes	4,732 (35.85)	4,321 (35.31)	411 (42.63)	
Cardiovascular disease				<0.001
No	11,563 (87.59)	10,754 (87.88)	809 (83.92)	
Yes	1,638 (12.41)	1,483 (12.12)	155 (16.08)	
DM				0.96
No	11,695 (88.59)	10,842 (88.60)	853 (88.49)	
Yes	1,506 (11.41)	1,395 (11.40)	111 (11.51)	
Dyslipidemia				1.00
No	9,103 (68.96)	8,438 (68.95)	665 (68.98)	
Yes	4,098 (31.04)	3,799 (31.05)	299 (31.02)	
Drinking				<0.001
No	8,782 (66.53)	8,070 (65.95)	712 (73.86)	
Yes	4,419 (33.47)	4,167 (34.05)	252 (26.14)	
Smoking				0.19
No	8,061 (61.06)	7,492 (61.22)	569 (59.02)	
Yes	5,140 (38.94)	4,745 (38.78)	395 (40.98)	

### 3.2 Hazard ratio of all-cause mortality death on tooth loss

During a median follow-up of 8.7 years, Cox regression models demonstrated a consistent and independent association between tooth loss and all-cause mortality, even after adjusting for potential confounders (Table 2). The multivariable-adjusted hazard ratio (HR) for tooth loss was 1.34 (95% CI: 1.08–1.67) in Model 3.

**Table 2:** Hazard ratio of all-cause mortality on tooth loss.

Group	Crude model	Model 1	Model 2	Model 3
Total sample	4.15 (3.36, 5.12)^#^	1.36 (1.09, 1.70)*	1.27 (1.02, 1.59)*	1.34 (1.08, 1.67)*
Age group				
45–59 y	2.27 (0.83, 6.21)	1.58 (0.57, 4.40)	1.58 (0.57, 4.38)	1.6 (0.58, 4.43)
60–79 y	1.71 (1.29, 2.26)***	1.14 (0.85, 1.52)	1.06 (0.79, 1.42)	1.14 (0.86, 1.53)
≥80 y	1.52 (1.03, 2.24)*	1.59 (1.07, 2.36)*	1.46 (0.98, 2.19)	1.62 (1.09, 2.41)*
Sex				
Female	3.78 (2.75, 5.20)^#^	0.95 (0.68, 1.33)	0.86 (0.61, 1.21)	0.9 (0.64, 1.26)
Male	4.71 (3.56, 6.23)^#^	1.8 (1.34, 2.41)	1.7 (1.27, 2.28)***	1.8 (1.34, 2.40)^#^
Residence				
Rural	3.39 (2.59, 4.44)^#^	1.17 (0.89, 1.55)	1.09 (0.82, 1.45)	1.16 (0.88, 1.54)
Urban	6.27 (4.47, 8.79)^#^	1.78 (1.24, 2.55)**	1.67 (1.16, 2.41)*	1.76 (1.22, 2.53)**

^#^ *p* < 0.0001.

*** *p* < 0.001.

** *p* < 0.01.

* *p* < 0.05.

Model 1 adjusted for sex, age, marital status and residence place; Model 2 adjusted for sex, age, marital status, residence place, BMI, drinking and smoking; Model 3 adjusted for sex, age, marital status, residence place, BMI, drinking, smoking, hypertension, DM, CVD, and dyslipidemia.

In a subgroup analysis, the association was stronger among older adults (≥80 years) with an HR of 1.62 (95% CI: 1.09–2.41) compared to younger participants. Moreover, males with tooth loss exhibited a significantly higher risk of all-cause mortality (HR: 1.80, 95% CI: 1.34–2.40) compared to females (HR: 0.90, 95% CI: 0.64–1.26). Similarly, individuals residing in urban areas had a higher risk of all-cause mortality (HR: 1.76, 95% CI: 1.22–2.53).

### 3.3 The casual mediation analysis of systematic inflammation on the relationship between tooth loss and all-cause mortality

The results showed that tooth loss had a significant direct effect on increasing all-cause mortality risk (*p* < 0.001). Additionally, serum CRP levels rather WBC counts mediated 3% of this effect, meaning that part of the benefit of tooth loss on all-cause mortality risk was due to its influence on elevated serum CRP level (*p* < 0.01) (Table 3).

**Table 3:** The casual mediation analysis of systematic inflammation on the relationship between tooth loss and all-cause mortality.

Systemic inflammation markers	ACME	ADE	Total effect	Proportion of mediated
CRP levels	0.003** (0.001, 0.01)	0.045*** (0.026, 0.07)	0.048*** (0.028, 0.07)	0.064 (0.016, 0.16)
WBC counts	−0.001 (−0.002, 0.000)	0.045*** (0.024, 0.07)	0.044*** (0.024, 0.06)	−0.015 (−0.050, 0.000)

** *p* < 0.01.

*** *p* < 0.001.

ACME, average causal mediation effect; ADE, average direct effect; CRP, C-reactive protein; WBC, white blood cell.

## 4 Discussion

The fully adjusted Cox proportional hazards model reveals that individuals with tooth loss have higher overall mortality rates compared to those without tooth loss. Recent study from Poland identified that factors such as female gender, lower education, higher BMI, higher fasting blood glucose, smoking, infrequent dental visits, and poor brushing habits were associated with individuals with losing more than 8 teeth (14). Tooth loss is also influenced by a complex interplay of factors such as marital status, place of residence, and alcohol consumption. Single women are more likely to experience disruptions in daily activities due to health issues, which may extend to poorer oral health maintenance and an increased risk of tooth loss. Urban-rural differences in access to dental services also contribute to disparities in oral health. For example, while this study identified higher rates of tooth loss among rural populations, a study from Japan revealed that individuals in rural areas were less likely to have a family dentist compared to those in urban areas (15). This limited access to dental care may result in a higher prevalence of untreated dental issues and tooth loss in rural communities. Alcohol consumption further complicates the relationship with tooth loss. Excessive alcohol intake has been associated with poor oral hygiene practices and an increased risk of periodontal disease (16), which can lead to tooth loss. Interestingly, however, this study has found that individuals who abstain from drinking were found to have a higher prevalence of tooth loss, suggesting that other confounding factors may play a role.

Growing evidence further confirms the bidirectional relationship between tooth loss and systemic conditions, including CVD, DM, hypertension, and even cancer (7). In this study, the Cox model has indicated that tooth loss increased the risk of mortality after adjusted CVD, DM, hypertension and dyslipidemia. Many chronic diseases are not only life-threatening but also significantly reduce life expectancy. Their prolonged progression of these diseases often leads to a gradual decline in bodily functions, increases the risk of comorbidities, and ultimately resulting in premature death.

CVDs, in particular, are a significant source of social and economic burden and represent a major global public health challenge (17). A recent systematic reviews and meta-analysis have confirmed an association between tooth loss and an increased risk of CVD mortality (18). Furthermore, a nationwide cohort study from Korea, which included 4,440,970 individuals, demonstrated a dose-dependent relationship between tooth loss and the incidence of myocardial infarction (MI), heart failure (HF), ischemic stroke, and all-cause mortality. For each missing tooth, the risk increased by approximately 1% for MI, 1.5% for both HF and stroke, and 2% for mortality. Tooth loss has proved to be a reliable predictor of cardiovascular outcomes (19). Similarly, a dose-response analysis indicated a 3% increase in the incidence of coronary heart disease (CHD) and stroke for every additional two lost teeth (17). Moreover, men with fewer than 24 teeth were found to have a higher risk of stroke compared to those with 25 or more teeth (20).

DM, the fourth leading cause of death globally, has also been shown to have a bidirectional cause-and-effect relationship with tooth loss (7, 21). A recent review of 13 studies found that tooth loss was associated with higher rates of DM and its complications, including heart disease, diabetic retinopathy, metabolic syndrome, and reduced quality of life (22). Additionally, a nationwide cohort study from revealed a link between tooth loss and elevated fasting glucose levels, while more frequent tooth brushing was found to lower fasting glucose levels (23). Similarly, another research from Finland reported that tooth loss was associated with impaired glucose metabolism in middle-aged adults (24). In addition, several cohort studies have found that individuals with more tooth loss had a higher incidence of hypertension during follow-up compared to those with fewer missing teeth (25).

Tooth loss can also lead to nutritional imbalances and negatively impact overall health. On the one hand, it causes masticatory dysfunction., and on the other hand, it alters food preferences (26). Studies have demonstrated that having fewer than 20 teeth, with or without dentures, results in longer chewing times, swallowing larger food particles, over-preparing and over-cooking meals, reduced intake of vegetables and fiber, increased fat and calorie consumption, and lower blood levels of vitamins and minerals (27). In addition, tooth loss can weaken the physical function in older adults, including muscle strength (grip strength) and physical performance (chair stand speed), which contributes to increased mortality (28). Another study also found that weight loss due to undernutrition functions as a mediator in the relationship between tooth loss and mortality (29).

In addition, systemic inflammatory mediates the association between tooth loss and mortality. C-reactive protein (CRP) is a blood marker commonly used to assess inflammation and infection. It is produced by the liver in response to inflammatory stimuli, with elevated levels typically indicating the severity of inflammation. The mediation analysis in this study highlighted the importance of systemic inflammation markers as a partial mediator in the relationship between tooth loss and all-cause mortality risk. This finding suggests that promoting oral health not only directly reduces all-cause mortality risk but also helps lower serum CRP levels, thereby further reducing the risk of mortality. Previous study has shown that tooth loss was associated with systematic inflammation markers (10). Specifically, individuals with 17–32 teeth lost tend to have higher concentration of CRP and WBC compared to those with 1–16 teeth loss (30), Recent study has also demonstrated that elevated *hsCRP* levels are independently associated with all-cause mortality after adjusting for a comprehensive range of lifestyle and clinical variables (31). Periodontitis, the leading cause of tooth loss, has been linked to elevated serum CRP levels (32, 33). High levels of hsCRP associated with periodontitis have been correlated with an increased prevalence of systemic diseases, including hypertension, DM and stroke (34), Similarly, another study demonstrated that systemic inflammation mediates the association between periodontitis and hypertension (35). Another major cause of tooth loss-dental caries has also been linked to the increased level of serum *hsCRP* (36). However, no significant mediator effect was detected in WBC count in this study, which warrants further investigation.

Oral microbiota might play an important role in the mediation of *hsCRP*. Tooth loss alters the distribution and structure of oral flora (7). The dysregulation of oral flora leads to the conversion of nitrate into nitrite, which is considered carcinogenic. CRP, as a biomarker of the innate immune response, is produced in response to inflammation triggered by exposure to oral bacteria (7). Poor oral health increases the risk of oral inflammation and oral flora disorder. Improving oral hygiene (10, 37) and restoring missing teeth (38) (e.g., through denture restoration or implantation) could decrease the disease-related mortality (38).

The present study has several potential limitations. Firstly, this retrospective cohort study studies followed for an average of only 8 years, which may not be sufficient to observe long-term effects, and longer follow-up periods are needed. Secondly, the diagnosis of tooth loss and several covariates, such as hypertension and DM, were based on self-reported data, which may lead to reporting bias. Finally, the study only assessed all-cause mortality, and no associations with cause-specific deaths were reported.

## 5 Conclusion

Our study shows that individuals with tooth loss exhibit higher mortality rates compared to those without tooth loss in the Chinese population, with serum hs-CRP identified as a mediator. Enhancing oral hygiene and restoring missing teeth might help reduce disease-related mortality.

## Data Availability

The original contributions presented in the study are included in the article/Supplementary Material, further inquiries can be directed to the corresponding authors.
